# Amendments before the Michigan Legislature

**Published:** 1903-03

**Authors:** 


### EDITORIALS.

### AMENDMENTS BEFORE THE MICHIGAN LEGISLATURE.

The following amendments have been offered to the bill pending before the Michigan Legislature. 1. That all persons appointed upon the Board must be graduates from colleges which are recognized by the A. V. M. A., residents of this State, and citizens of the United States. 2. That all colleges having a curriculum of three years of six months each, and conducted under the rules of the A. V. M. A., shall be recognized, and their graduates shall be registered upon presentation of a diploma which has been issued to the applicant.

We are informed that those amendments were opposed by some of the veterinary profession of Michigan, and we are at a loss to understand why this should be. If the profession of Michigan are still of the opinion that aliens should be allowed to register without becoming citizens of the United States, and hold positions on State Boards, we are quite sure that they will find the profession of the United States a unit against such a state of affairs. If they are opposed to the standard of qualifications adopted by the A. V. M. A. for its membership requirements they are equally opposed to higher veterinary education, and the profession of this country are entitled to know what the Michigan veterinarians stand for.

